# Direct and convenient measurement of plasmid stability in lab and clinical isolates of *E. coli*

**DOI:** 10.1038/s41598-017-05219-x

**Published:** 2017-07-06

**Authors:** Siyi Chen, Mårten Larsson, Robert C. Robinson, Swaine L. Chen

**Affiliations:** 1 Department of Medicine, Yong Loo Lin School of Medicine, National University of Singapore, 1E Kent Ridge Road, NUHS Tower Block, Level 10, Singapore, 119228 Singapore; 2 Institute of Molecular and Cell Biology, A*STAR (Agency for Science, Technology and Research), 61 Biopolis Drive, Singapore, 138673 Singapore; 3 GERMS and Infectious Diseases Group, Genome Institute of Singapore, 60 Biopolis Street, Genome, #02-01, Singapore, 138672 Singapore; 4 Department of Biochemistry, National University of Singapore, Singapore, 117597 Singapore; 5 NTU Institute of Structural Biology, Nanyang Technological University, 59 Nanyang Drive, Singapore, 636921 Singapore; 6 Research Institute for Interdisciplinary Science, Okayama University, Okayama, 700-8530 Japan; 7 Present Address: Department of Medical Biochemistry and Microbiology, Uppsala University, SE-751 23 Uppsala, Sweden

## Abstract

Plasmids are important mobile elements in bacteria, contributing to evolution, virulence, and antibiotic resistance. Natural plasmids are generally large and maintained at low copy number and thus prone to be lost. Therefore, dedicated plasmid maintenance systems have evolved, leading to plasmid loss rates as low as 1 per 10^7^ divisions. These low rates complicate studies of plasmid loss, as traditional techniques for measuring plasmid loss are laborious and not quantitative. To overcome these limitations, we leveraged a stringent negative selection system to develop a method for performing direct, quantitative measurements of plasmid loss in *E. coli*. We applied our method to gain mechanistic insights into a heterologously reconstituted segregation system in lab strains and clinical isolates of *E. coli*. We also performed direct stability studies of a currently circulating resistance plasmid in a clinical isolate, strain EC958, which is a member of the rapidly expanding global ST131 *E. coli* clone. Our results establish the foundational assays required to screen for small molecules targeting plasmid stability, which could complement current strategies for reducing the spread of antibiotic resistance, complementing other strategies for treating antibiotic resistant bacteria.

## Introduction

Plasmids are extrachromosomal genetic elements carried by many bacteria^1,2^. Basic insights into plasmid biology have been transformative for molecular biology and biotechnology. In their natural context, plasmids are evolutionarily important as mobile elements that allow rapid, wholesale gene gain and loss within and between species via horizontal gene transfer^3,4^, through both conjugative and nonconjugative mechanisms^2^. In pathogenic bacteria, plasmids are often important for virulence and commonly carry antibiotic resistance genes^1–3,5^. The facile transmission of multiple resistance genes on a single plasmid contributes to the clear and present public health problem of rising antibiotic resistance rates^6,7^. Two recent notable examples highlighting the dangers of plasmid-borne antibiotic resistance are the rapid spread across the globe of the New Delhi metallo-β-lactamase-1 plasmid (pNDM-1, carrying the *bla*_*NDM*-1_ β-lactamase gene)^8^ and several related sequence type 131 (ST131) plasmids (including pEK499 and pEC958)^9,10^, which carry up to 10 resistance genes, including genes encoding extended spectrum beta-lactamases^11–13^. These plasmids and the resistances they confer are key contributors to the success of their original *E. coli* hosts^14^ and, worryingly, are able to spread to related pathogens such as *Klebsiella pneumoniae* and *Acinetobacter baumanii*^15^. This pattern of rapid antibiotic spread through plasmids continues to repeat itself, with the recent discovery of the plasmid-borne *mcr-1* gene conferring resistance to the last-resort drug colistin^16^. Despite the central role of plasmids in antibiotic resistance, no current therapeutic molecules are available that block plasmid maintenance and spread. Therefore, basic knowledge of plasmid maintenance, stability, and transmission has high relevance both for treatment of individual patients and for public health, where directly targeting plasmids represents a much needed novel control strategy.

Naturally occurring conjugative plasmids such as those carrying the *bla*_*NDM-1*_ or *mcr-1* genes or those found in the clonally-related set of clinical isolates collectively referred to as ST131 are typically large (>50–100 kb) and present at low copy number (1–2 per cell)^2^. They therefore rely on several plasmid maintenance systems to ensure their propagation. Targeting any of the maintenance systems might reduce plasmid stability and thereby provide a strategy to decrease population rates of antibiotic resistance. Plasmid maintenance systems include replication, copy number control, multimer resolution, partitioning/segregation, post-segregational killing, and direct horizontal transfer (such as conjugation) systems^17–19^. One well studied plasmid segregation system is the Type II *par* locus from plasmid R1^17^, which was originally isolated from *Salmonella paratyphi*^20^. In brief, the R1 segregation locus, collectively referred to as *parCMR*, consists of three genetic elements. The *parC* (centromeric) element consists of a promoter that drives expression of the *parM* and *parR* genes. In addition, it contains binding sites for the ParR protein. The *parM* (motor) gene encodes the actin-like ParM protein that can polymerize to form filaments. The *parR* (repressor and adapter) gene encodes the DNA-binding ParR protein. Two copies of a plasmid carrying the *parCMR* locus are each bound by ParR proteins at their *parC* loci. ParM in turn binds to the *parC*-bound ParR proteins; insertional polymerization of ParM leads to physical pushing of the two plasmid copies to opposite ends of the cell. Upon cell division, each daughter cell then inherits one of the plasmid copies^17^.

To study plasmid maintenance systems, a convenient assay for plasmid loss is essential. Unfortunately, despite the ease of detecting the *presence* of a plasmid by using antibiotic resistance markers, detecting *loss* of a plasmid is laborious, requiring manual replica plating or patching of hundreds or thousands of colonies to find the minority of cells which have lost the resistance marker^21^, limiting the sensitivity for detecting very small proportions of plasmid-free cells. Screenable phenotypes such as those based on LacZ expression are more convenient, but they still suffer from limited practical sensitivity for quantifying a minor population of bacteria below approximately 0.01% of the total.

Another drawback of traditional plasmid loss methods is that they measure plasmid loss frequencies and not plasmid loss rates. Luria and Delbrück clarified this distinction for the occurrence of spontaneous mutations in bacteria that confer phage resistance^22^; mutation rate is typically the desired parameter to be measured, but most laboratory assays measure frequency (or number of mutant bacteria that are resistant to phage within a given population). Rates and frequencies can be quite different if a mutation arises very early in the growth of a bacterial culture, leading to an exponentially increasing number of mutant descendents (a so-called jackpot effect). Modeling of results from multiple replicate cultures (all of which individually measure mutation frequencies) can be used to infer the actual mutation rate. In our study, mutation to phage resistance can be equated with plasmid loss. A novel system for directly detecting plasmid loss, based on an engineered negative selection system, has recently been reported which eases the study of plasmid stability by enabling accurate measurements of plasmid loss rates (instead of frequencies) using a modified Luria-Delbrück fluctuation test^23^. However, this system requires matched modifications of both the plasmid and chromosome and has only been used in a cloning strain of *E. coli*, limiting its utility for medically relevant wild type isolates carrying resistance plasmids of current clinical concern.

Recently, our lab has developed a set of powerful negative selection systems for use in unmodified clinical isolates of Enterobacteriaceae^24^. In this work, we have utilized one of our negative selection cassettes to develop a method for performing direct, quantitative measurements of plasmid loss in *E. coli*. The approach can be readily applied to other pathogenic bacteria, enabling convenient studies of how medically relevant plasmids are maintained, with a particular advantage of enabling quantitative and mechanistic studies directly in clinical isolates.

## Results

**Figure 1:**
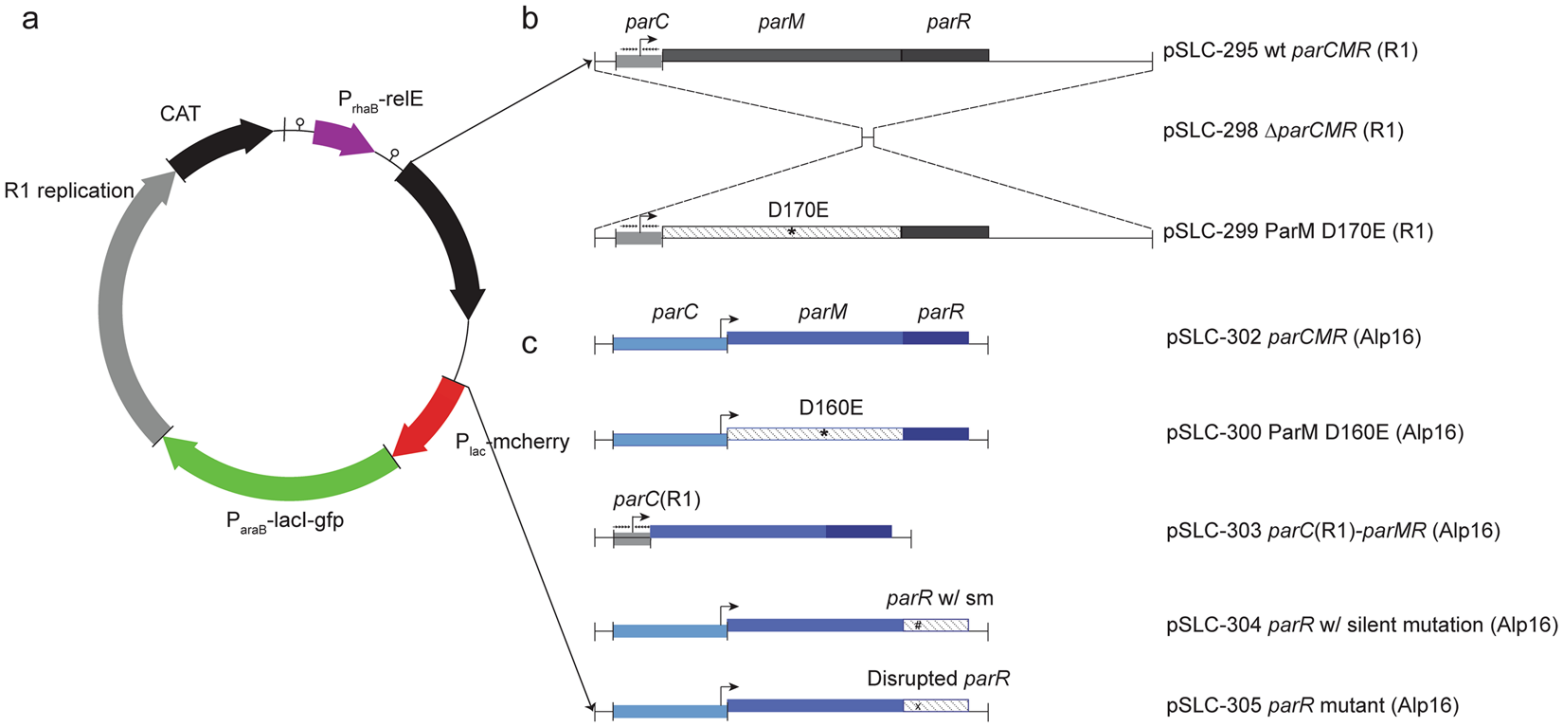
Test plasmid utilizing negative selection for quantitative measurement of plasmid loss. (**a**) Genetic map of a synthetic plasmid for studying isolated plasmid stability elements. Genetic modules are depicted as thick arrows, which include both the promoter and gene body. Vertical bars (|) represent unique restriction digestion sites, and a small circular symbol represents a transcriptional terminator. (**b**) Genetic mutants used to examine the mechanism of the R1 *parCMR* segregation system. Genetic elements are depicted as grayscale rectangles, with genes above or below the central line corresponding to the coding strand. The promoter within *parC* is indicated by a bent arrow. Smaller horizontal arrows indicate sequence repeats within *parC* that are important for ParR binding. An asterisk indicates a point mutant within *parM*. (**c**) Genetic mutants used to examine the function and mechanism of the *Clostridium perfingens* Alp16 *parCMR* segregation system. All Clostridial genes are depicted in shades of blue, with symbols as in (**b**).

We created a test plasmid to characterize the utility of our negative selection system for plasmid inheritance studies. This synthetic plasmid (Fig. 1a), carrying the negative selection cassette in addition to several features convenient for validating its presence and loss (including a positively selectable antibiotic marker conferring chloramphenicol resistance; two inducible fluorescent markers (mCherry under the control of the Lac promoter; GFP under the control of the arabinose promoter); and unique restriction sites for insertion of additional modules), was synthesized with the replication origin and segregation system from the *E. coli*/*Salmonella* R1 plasmid^25^. This plasmid was maintained in a cloning strain of *E. coli*, in which it conferred the expected chloramphenicol resistance and inducible mCherry (by IPTG) and GFP (by arabinose) expression.

Our negative selection system is based on tightly regulated expression of the *relE* toxin. Under permissive conditions, *relE* is not expressed and cells grow normally, while under restrictive conditions (minimal media supplemented with rhamnose to induce expression from the P_*rhaB*_ promoter), the *relE* gene is expressed, leading to growth arrest via cleavage of translating mRNAs at the A-site of the ribosome^26^. Cells that do not carry the plasmid, however, can grow normally under these restrictive conditions, leading to a simple selection for cells that have lost the plasmid. Of note, other processes besides plasmid loss, such as mutation or deletion of the negative selection cassette, can enable bacteria to grow under restrictive conditions, but these may be distinguished by testing for chloramphenicol resistance or mCherry fluorescence.

**Figure 2:**
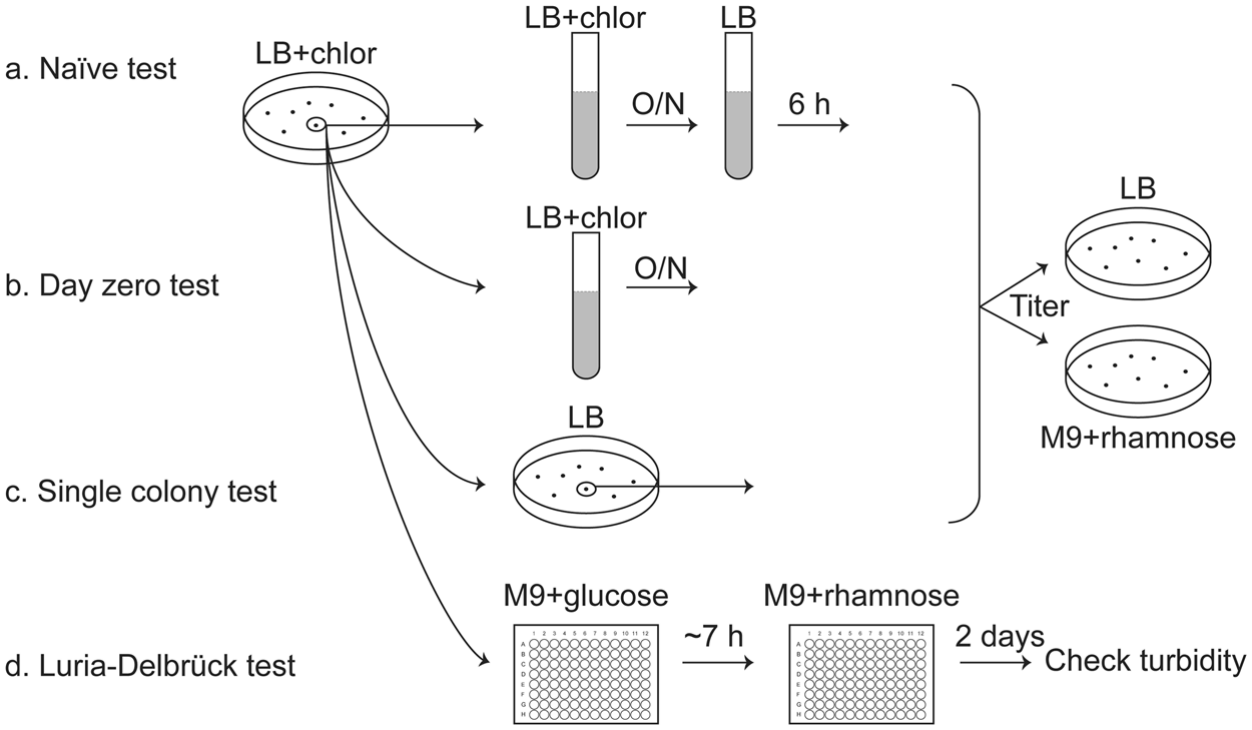
Schematic of the plasmid stability tests. Depicted are the key differences between the naïve (**a**), day zero (**b**), single colony (**c**), and Luria-Delbrück (**d**) tests. Of these, the first three are loss frequency tests, while the Luria-Delbrück assay is a loss rate test.

**Figure 3:**
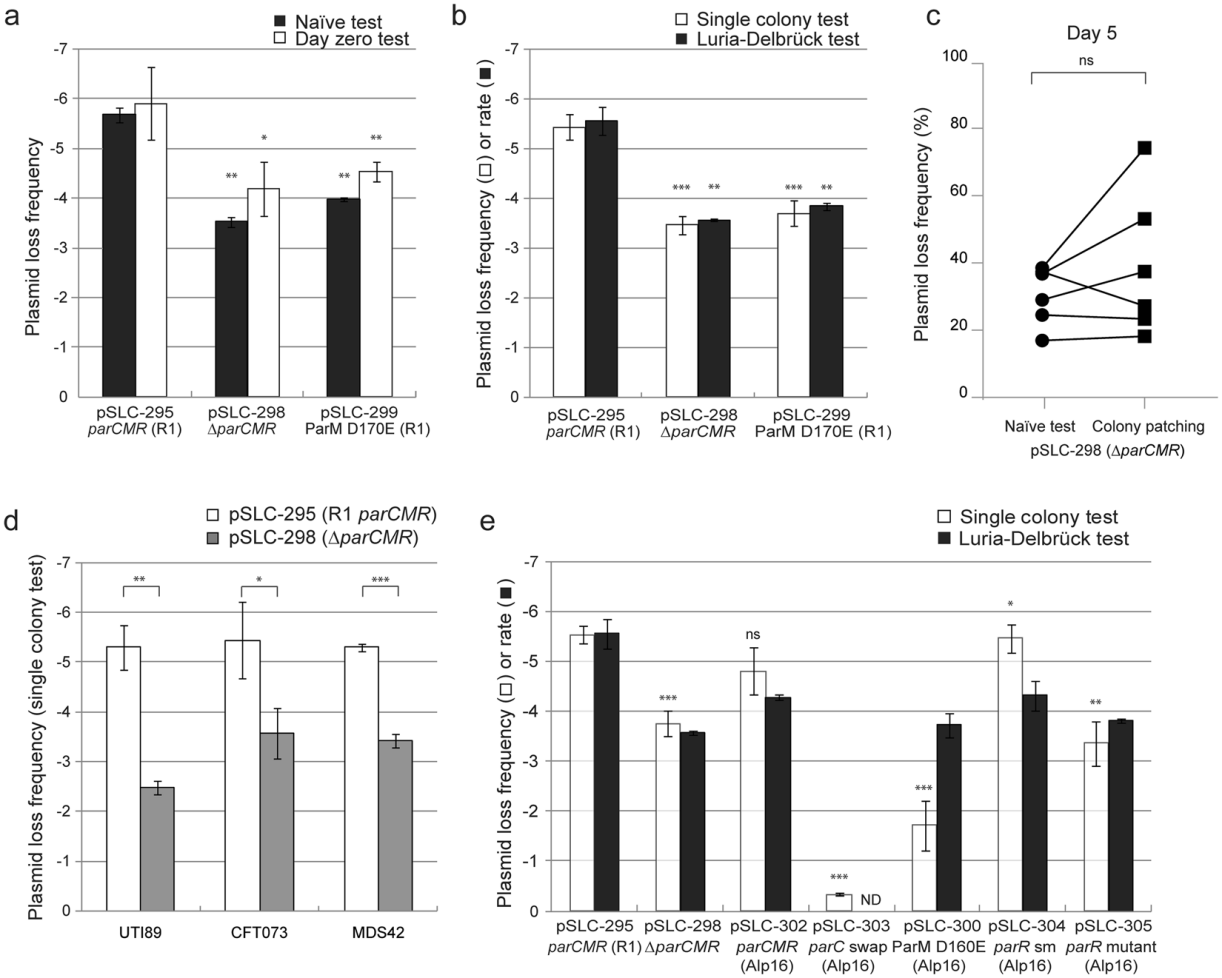
Contribution of *parCMR* systems to plasmid stability. Plasmid loss frequencies and rates for test plasmids carrying *E. coli/Salmonella* R1 *parCMR* mutations in the lab strain MDS42 determined using the naïve and day zero tests (**a**) and the single colony and Luria-Delbrück tests (**b**). Significant differences from the plasmid loss frequency of pSLC-295 were tested using a 2-tailed Student’s t-test on log transformed values. ns indicates p > = 0.05; *p < 0.05; **p < 0.01; ***p < 0.001. (**c**) Comparison of plasmid loss frequencies as measured by the naïve test and patching of individual colonies. MDS42 carrying plasmid pSLC-298 (*ΔparCMR*) was passaged without antibiotics for 5 days (n = 3 for each of 2 independent biological experiments). The same culture was assayed for plasmid loss frequency by both tests; results for the same culture are indicated by a connecting line between the data points. There was no significant difference (p = 0.3125, Wilcoxon signed rank test) in measured loss frequencies between the tests. (**d**) Measurement of test plasmid stability in clinical isolates (UTI89 and CFT073) compared with those measured in a lab strain (MDS42). Significant differences between pSLC-295 and pSLC-298 for each host strain are calculated and indicated as in panels (a) and (b). (**e**) The *Clostridium perfringens* Alp16 *parCMR* system functions similarly to the R1 *parCMR* system. Plasmid loss tests for MDS42 cells carrying plasmids with mutants of the Alp16 *parCMR* system (indicated on the x-axis) using the single colony and Luria-Delbrück tests. Significant differences are calculated and indicated as in panels (a) and (b). Comparisons for pSLC-298 and pSLC-302 are against pSLC-295. Comparisons for pSLC-303, pSLC-300, pSLC-304, and pSLC-305 are against pSLC-302. ND, not determined. For all graphs, plasmid loss frequency data are plotted as the mean of log-transformed values from at least three independent experiments with error bars indicating standard deviations, while the Luria-Delbrück rate data are plotted as the mean of log-transformed values from at least two independent experiments with error bars depicting the range between the two values. Significance was tested for differences in Luria-Delbrück rates only when > = 3 experimental replicates were performed.

A simple test for plasmid loss (which we refer to as the “naïve” test; Fig. 2a) is to plate equal numbers of bacteria on permissive and restrictive conditions and measure the fraction of colony forming units that grow under restrictive conditions. To ensure that bacteria are carrying the plasmid initially, they are first maintained in the presence of chloramphenicol and then grown for 4–6 hours without antibiotic to allow plasmid loss to occur. This naïve test yielded 1 CFU under restrictive conditions per 10^6^ bacteria plated (a loss *frequency* of 10^−6^) (pSLC-295 wt R1 *parCMR*; Fig. 3a). To ensure that colonies growing under restrictive conditions had indeed lost the plasmid, we patched them onto LB/IPTG (to induce mCherry fluorescence), LB/arabinose (to induce GFP fluorescence), and LB/chloramphenicol (to test for the resistance gene). All of the colonies growing on rhamnose that we tested (20/20) had lost GFP and mCherry fluorescence as well as chloramphenicol resistance, indicating they had indeed lost the plasmid instead of inactivating the negative selection cassette by mutation. These data are consistent with the high reported stringency (3 × 10^−8^/CFU) of the negative selection cassette^24^, which indicated that mutation or deletion of the cassette is rare in *E. coli* and *Salmonella*.

As an example of the value of this system for understanding plasmid maintenance mechanisms, we used the ability to select for plasmid-free cells to validate the function of individual elements in the R1 *parCMR* segregation system (Fig. 1b). Furthermore, we could also measure the quantitative contribution of this segregation system to plasmid stability. Deletion of the entire *parCMR* locus (pSLC-298, ∆*parCMR*) led to a loss frequency of 10^−4^; this 100× increase in stability due to *parCMR* is in excellent agreement with previous reports of the wild type R1 plasmid^25^, but in absolute value 10- to 100-fold lower than previously measured for a synthetic mini-R1 replicon^23^. Importantly, the increase in loss frequency was not associated with lower plasmid copy number (which could also increase plasmid instability) (Table S1) and the carriage of these plasmids had no detectable effects on bacterial growth rates (Figure S1a). Furthermore, mutation of a catalytic residue for the motor protein ParM ATPase (pSLC-299, ParM D170E)^27^ also led to a similar 100× increase in loss frequency (Fig. 3a). As an additional control, to ensure that the plasmid loss measured on rhamnose was truly due to ongoing loss during bacterial growth, cells taken directly from culture in chloramphenicol were plated immediately onto rhamnose (denoted the “day zero” test; Fig. 2b). The expectation was that only a background level (3 × 10^−8^) of colonies would grow, as all viable bacteria taken directly from chloramphenicol are expected to carry the plasmid; instead, we found that the number of plasmid-free cells correlated with the loss frequency measured by the naïve test (Fig. 3a).

To examine this unexpected correlation, we performed a quantification of plasmid loss *rates* using a modified Luria-Delbrück test (Fig. 2d)^22,23^. The Luria-Delbrück test demonstrated a 100× increase in plasmid loss rate when the *parCMR* segregation system was deleted (pSLC-298; Fig. 3b), validating the results from the naïve test. However, the Luria-Delbrück test is inconvenient due to its requirement for a series of identical cultures (50–100), whose starting size must be tuned to minimize pre-existing mutants (plasmid-free cells) in the starting culture^21^. We therefore devised a fourth test (the “single colony” test; Fig. 2c) to ensure that 100% of the starting bacteria indeed carried the plasmid. Bacteria were streaked to single colonies in the absence of antibiotics; each colony in this case results from the growth of a single founder bacterium, which must either carry the plasmid or be plasmid-free (i.e. the colony starts as 100% plasmid-containing or 100% plasmid-free). We titered an entire single colony on both chloramphenicol and on rhamnose; the presence of any colonies on chloramphenicol verified that the original single founder must have carried the plasmid. The colonies growing on rhamnose could then be concluded to have truly lost the plasmid (instead of arising from a founding plasmid-free bacterium). In the single colony assay, deletion of the R1 *parCMR* system again resulted in a 100× increase in plasmid loss frequency (Fig. 3b).

As an additional demonstration of the enhanced accuracy and convenience of our negative selection system, we compared plasmid loss frequencies as measured by the naïve test with the traditional technique of patching many colonies. Given the low loss frequency for pSLC-295 (10^−6^) and pSLC-298 (10^−4^) in the naïve test, as expected, we found no antibiotic sensitive colonies among 100 patched colonies of strains carrying either of these plasmids after 1 day of passage without antibiotics. We therefore passaged the cultures without antibiotic selection once per day for 5 days. We again were unable to find any antibiotic sensitive colonies among 100–300 patched colonies of bacteria carrying pSLC-295, consistent with a loss frequency after 5 days of 0.2–4.9 × 10^−3^ as measured by the naïve test. Furthermore, in two independent experiments, each with three replicate cultures, we found that there was no significant difference in the quantification of plasmid loss frequency by patching or by the naïve test for bacteria carrying pSLC-298 after 5 days (16.8–38.5% for the naïve test; 18.2–74.2% by patching 200–500 colonies per culture; p = 0.3125, Wilcoxon signed-rank test; Fig. 3c).

We verified that these plasmid loss assays could be recapitulated using two clinical isolates of *E. coli*: UTI89, a cystitis UPEC strain^28^; and CFT073, a bloodstream isolate of a pyelonephritis UPEC strain^29^. Quantitative stability measures were in good agreement for the same plasmid in different strains, and the *parCMR* system also contributed 2–3 orders of magnitude to maintenance fidelity in these clinical UPEC strains (Fig. 3d). These similar results in clinical isolates indicates that our negative selection system enables plasmid loss studies directly in clinical disease-causing Enterobacterial isolates.

To demonstrate the power of this system for understanding novel plasmid loci contributing to stability, we used the synthetic plasmid to study a predicted plasmid segregation system (Alp16 *parCMR*) from a very distantly related bacterium, *Clostridium perfringens* (Fig. 1c). Despite the large phylogenetic distance of *C. perfringens* from *E. coli*, the Alp16 *parCMR* system did indeed confer enhanced stability to the synthetic plasmid (pSLC-302), which was dependent on both functional *parM* and *parR* genes coupled with the *parC* centromeric binding site (Fig. 3e; see Supplemental text for full details including expression control of the Alp16 *parCMR* system). We again found good correlation between the single colony and Luria-Delbrück tests, except when the mutations introduced caused differences in the growth rates of plasmid-containing and plasmid-free cells (faster growth of plasmid-free cells leads to an overestimate of plasmid loss frequency; Figure S1b). Therefore, our synthetic plasmid platform, featuring a convenient and stringent negative selection marker, provided *in vivo* functional validation of all components of a previously unstudied *parCMR* system, making it a promising platform for both identification and characterization of other unstudied plasmid maintenance systems.

**Figure 4:**
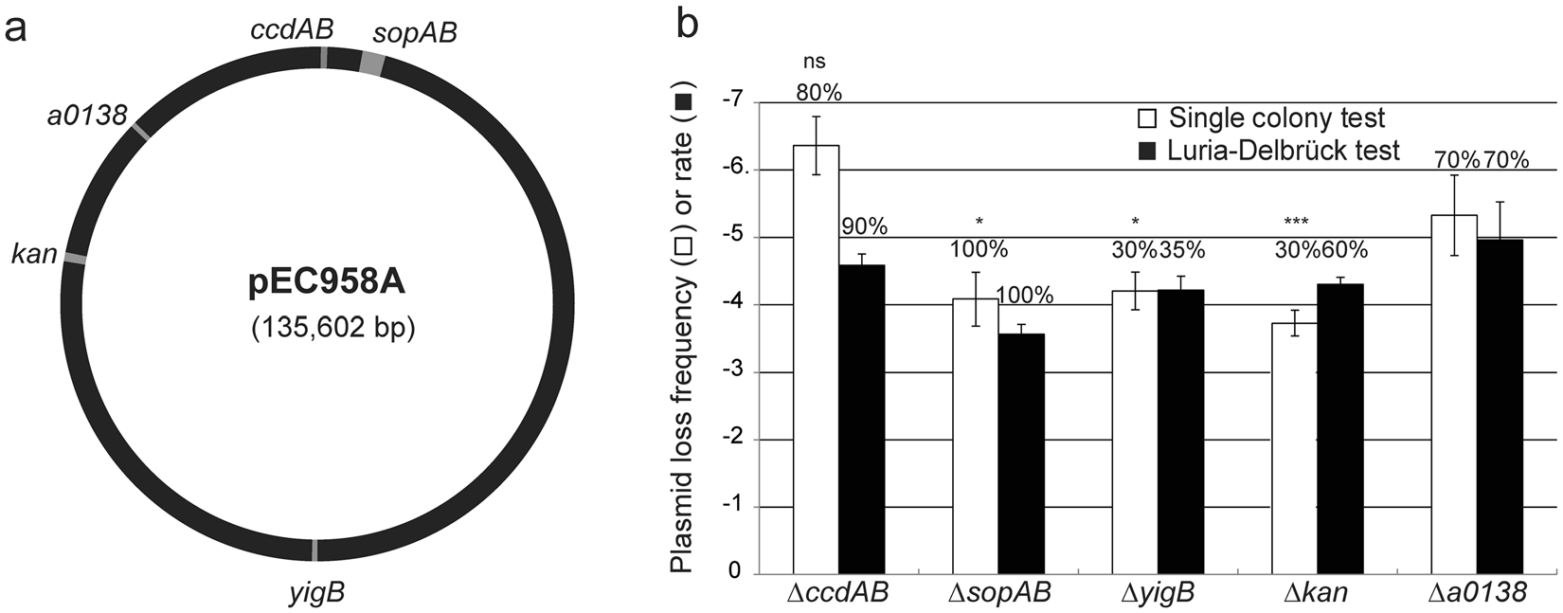
Direct measurement of pEC958 plasmid stability in its native host strain, EC958. (**a**) Genetic map of plasmid pEC958 indicating the location of genes (highlighted in gray) replaced by the negative selection cassette. (**b**) Plasmid stability measured in EC958. Raw plasmid loss frequencies (assayed using the single colony test, white bars) or rates (assayed using the Luria-Delbrück test, black bars) are plotted with error bars as in Fig. 3. Numbers above each bar indicate the percentage of colonies growing that are truly plasmid-free based on antibiotic resistance and PCR for plasmid-specific sequences. Significant differences in plasmid loss frequency were calculated relative to pSLC-344 (pEC958 *∆a0138*) using a 2-tailed Student’s t-test on log transformed values. ns indicates p > = 0.05; *p < 0.05; ***p < 0.001. Plasmid loss frequency data are plotted as the mean of log-transformed values from at least three independent experiments with error bars indicating standard deviations, while the Luria-Delbrück rate data are plotted as the mean of log-transformed values from at least two independent experiments with error bars depicting the range between the two values. Significance was tested for differences in Luria-Delbrück rates only when > = 3 experimental replicates were performed.

Antibiotic resistance plasmids of current medical concern might be more complex to study than a synthetic plasmid system. Examples of such complexities include multiple replication origins or recombinogenic mobile elements^9^. The ability to study such plasmids, directly in their native hosts (disease-causing clinical isolates) and with minimal modifications, would enable characterization of the contributions of these more complex features to plasmid stability. Furthermore, from the perspective of developing small molecules to disrupt maintenance of these antibiotic resistance plasmids, being able to test them directly in their native host strains would be a distinct advantage. We tested the negative selection cassette in plasmid pEC958, a large IncF plasmid encoding 6 resistance determinants, that is representative of plasmids carried in 97% of ST131 isolates globally^9^. Transposon-directed insertion sequencing had previously identified genetic elements qualitatively required for pEC958 maintenance, though quantification of the contribution of each locus to stability was not possible. Initial experiments validated that the negative selection cassette was usable in EC958, with baseline stringency in the chromosome similar to that measured in UTI89 (6 × 10^−8^ CFU growing on rhamnose per CFU plated). We inserted the cassette in multiple loci in pEC958, disrupting 2 loci important for pEC958 stability and 3 others that were not necessary (Fig. 4a). Both the single colony and Luria-Delbrück tests verified that the *sopAB* segregation system provided an additional ~30× stability to pEC958 when compared with the *a0138* (a gene of unknown function that is qualitatively not required for plasmid stability) disruption (although there was a defect in growth when the cassette was inserted into *sopAB*; Figure S1c). Interestingly, removal of the *ccdAB* toxin-antitoxin system seemed to slightly increase stability by the single colony test, but this was not statistically significant. Indeed, by the more accurate Luria-Delbrück test, it had only a minor effect (~3×) on the stability of pEC958; this is likely because we disrupted the entire operon instead of just the antitoxin as was previously reported^9^. Plasmids carrying the negative selection cassette in two other loci not required for pEC958 stability, *yigB* and the aminoglycoside resistance gene, were ~10× less stable than the *a0138* disruption (Fig. 4b). Because these two genes are in a region of pEC958 that contains insertion sequences that can mediate high frequency rearrangements, we tested for true plasmid loss by assaying for plasmid-encoded antibiotic resistance (tetracycline and kanamycin), which also correlated with a PCR test for the *oriV-1* plasmid origin (Figure S2). Compared with the other three loci, the negative selection cassette was lost more frequently despite overall plasmid maintenance when it was inserted into *yigB* or *kan*, likely due to inactivation or deletion by recombination of nearby insertion sequences (Fig. 4b).

## Discussion

Plasmids are foundational tools for biotechnology and important agents in bacterial evolution and antibiotic resistance. An understanding of the basic biology of plasmids is required for improved applications in both of these areas. One of the fundamental functions of a plasmid is to ensure its own reliable propagation. This is achieved through positive transmission (vertically via replication origins and segregation systems; horizontally via conjugation systems) and negative maintenance (killing of plasmid-free cells via toxin-antitoxin systems). The study of these maintenance functions relies on identification of both plasmid-carrying and plasmid-free cells. Traditionally, antibiotic markers made the identification of plasmid-carrying cells relatively easy. The identification of plasmid-free cells, however, especially with wild type plasmids whose maintenance is very efficient, typically required screening many individual colonies^21^; matched genetic modification of the plasmid and the host chromosome^23,30^; or propagation over extended periods to increase the fraction of plasmid-free cells^31,32^. We have surmounted all of these problems with the use of a recently published, efficient negative selection system^24^. Furthermore, we have developed several convenient assays for measuring both plasmid loss frequencies and rates and validated their utility in both lab and clinical strains.

The use of manual replica plating, or of amplification of plasmid-free cells by extended passage, introduces quantitative uncertainty into the measurements of low plasmid loss rates. For very stable plasmids, this therefore requires extended culture periods to enhance the loss frequency, which can further amplify the variation due to the “jackpot” effect of early mutations^22^. The only previous solution to this problem was to use matched plasmid and chromosomal genetic systems that would enable direct identification or selection of plasmid-free cells^23,30^, which then enabled the application of the Luria-Delbrück fluctuation test to measure plasmid loss rates^23^. Of note, our system estimates plasmid loss frequencies similar to the traditional patching technique when loss rates are reasonably high after extended culture. In addition, our system can measure plasmid loss at earlier time points and lower loss rates; these measurements at lower loss rates are both internally consistent and correspond with previously published results^25^. Finally, our negative selection system now enables the facile generalization of these experimental and quantitative benefits to the study of both synthetic and natural plasmids in lab strains and clinical isolates.

The Luria-Delbrück test is the most accurate of the plasmid loss tests we and others have used^23^; however, having an efficient selection system for plasmid-free cells enables several more convenient assays, particularly the naïve, single colony, and day zero tests. While a correlation in plasmid loss frequencies measured by the naïve and single colony tests was expected, we noted that these also correlated with the loss frequency measured by the day zero test. In the day zero test, bacteria are plated to rhamnose directly from a culture containing antibiotics (after washing to remove residual antibiotics and other sugars from the media); in principle, we expect all cells to contain the plasmid. One general possibility is that there are indeed plasmid-free cells in the antibiotic culture, which may survive in a persister-like state until they are plated on rhamnose; however, the rate of persister cell formation is typically quite low (10^−5^)^33^. Given the number of plasmid-free cells generated during typical cultures (as high as 10^−2^–10^−3^ for some plasmids tested), this seems to be quantitatively unreasonable to generate the number of surviving plasmid-free cells we identified. Furthermore, persister formation would also not explain the correlation with plasmid loss rates as measured by the Luria-Delbruck test. Instead of persisters, plasmid-free cells could also be generated by plasmid loss in the most recent cell divisions prior to plating on rhamnose. Cells that recently lost the plasmid will not generally be killed immediately because of the time of action required by antibiotics to kill^34,35^ and because these cells still will contain some level of the chloramphenicol acetyltransferase protein immediately after plasmid loss. A third alternative is that that there actually are no plasmid-free cells in the antibiotic culture; this could be possible if, upon plating on rhamnose, those cells that lose the plasmid in the first (one or more) cell division(s) are the ones that grow. Both of the latter two explanations would explain the correlation of the measured plasmid loss frequency in the day zero test with the other assays. In addition, both of these latter two possibilities imply that the day zero test is actually measuring plasmid loss over a limited number of generations, thus better approximating a plasmid loss rate test.

One of the more useful conceptual aspects of plasmids is their inherent modularity, both as autonomously replicating genetic elements as well as at the level of their constitutive genetic components. For the most part, plasmids are accurately described as the sum of their parts; for example, modern cloning plasmids contain a replication origin, an antibiotic resistance marker, and a “payload” region often containing a multiple cloning site. This extends to plasmid maintenance systems, and it has long been recognized that segregation systems, for example, appear to be self-contained genetic units that might be mixed and matched successfully with other plasmid components^36^. Indeed, we took an explicitly modular approach in constructing the synthetic plasmid pSLC-295, and further extended the concept by introducing the heterologous *C. perfringens* Alp16 *parCMR* system. Introduction of plasmid components found in such a distantly related bacterium (trivially, *C. perfringens* is Gram positive with <30% GC content^37^, while *E. coli* is Gram negative with ~50% GC content^38^) requires attention to several issues for expression control, which are discussed in detail in the Supporting Information, with accompanying data in Figure S3 and Table S2. We note that while other segregation systems from bacteria as diverse as chromosomal *parABS* systems from *Pseudomonas putida*^39^, *Bacillus subtilis*^40^, and *Burkholdera cenocpacia*^41^ and the plasmid pSK41 *parCMR* system from *Staphylococcus aureus*^32^ have been reconstituted in *E. coli*, each of these previous studies utilized known promoters directing expression in *E. coli*, often with a two-plasmid system, to obviate one or more of these issues. Our study is the first to directly take the entire *parCMR* system (only changing codon usage) and demonstrate its function as a single genetic unit. Further, we are the first to demonstrate by targeted mutagenesis that all three genetic components (*parC*, *parM*, and *parR*) are required for segregation, and therefore are likely functioning as we have predicted by homology from the R1 system. These results were enabled by our ability to accurately quantify plasmid loss rates.

In summary, the application of an improved negative selection cassette has enabled us to perform direct, quantitative studies of plasmid maintenance systems both on synthetic plasmids in cloning strains of *E. coli* and on currently circulating antibiotic resistance plasmids in their natural infectious host strains. The ability of plasmids to confer resistance to multiple antibiotics, ensure their stable maintenance, and transmit to other strains via conjugation or transformation make plasmids particularly bad actors in modern medicine’s current crisis of antibiotic resistance^3,6^. However, despite their high value as a target, no current therapies directly target plasmid stability. We posit that this is due to the lack of convenient tools for basic studies of plasmid maintenance, particularly those enabling precise quantification^21,23^. This lack of tools severely hampers the ability to search for potential compounds that would directly affect plasmid maintenance (by inhibiting plasmid replication or segregation, for example). While *in vitro* screens for isolated maintenance systems could be devised, these would suffer from known hurdles for cell penetration and would furthermore lack good *in vivo* tools for demonstrating efficacy in reducing the stability of wild type plasmids within wild type clinical isolates. Therefore, a direct, quantitative, cell-based, screenable assay usable in clinical isolates would be ideal. The synthetic plasmid pSLC-295 demonstrates how a customized *in vivo* system could be designed to target individual maintenance systems, while the application to pEC958 demonstrates that both primary screens and subsequent validation assays are also enabled by this technology. Thus, we have now surmounted the key practical barriers to developing drugs for a previously inaccessible class of therapeutics.

## Methods

### Strains, plasmids, and primers

All strains and plasmids used in this study are listed in Table S3. Plasmid pSLC-295 was synthesized in its entirety by GenScript (Piscataway, NJ, USA). Plasmids were electroporated into *Escherichia coli* K-12 strain MDS42 or clinical *E. coli* isolates UTI89, CFT073, or EC958 to yield strains SLC-726, SLC-729 to SLC-731, SLC-733 to SLC-736, SLC-896 to SLC-899, and SLC-908. All primers used in this study are listed in Table S4.

### Media and culture conditionsh

All bacteria were grown in LB or M9 media. Antibiotics and concentrations used were chloramphenicol (20 μg/mL), gentamicin (2 μg/mL), kanamycin (50 μg/mL), and tetracycline (10 μg/mL), unless otherwise specified. M9 medium was supplemented with either 0.2% glucose to suppress the toxic gene (*relE*) expression or 0.2% rhamnose for *relE* induction. In experiments where mCherry expression was induced, IPTG (isopropyl β-D-1-thiogalactopyranoside) was added at a final concentration of 1 mM. In experiments where GFP expression was induced, arabinose was added at 0.2%. All strains were streaked out on agar plates for single colonies and incubated at 37 °C. All broth cultures were inoculated from single colonies from these agar plates and incubated at 37 °C in a shaking incubator at 200 rpm.

### Design of pSLC-295

Plasmid pSLC-295 was synthesized using sequences extracted from Genbank. The sequence of the *E. coli/Salmonella* plasmid R1 origin of replication, *oriR1*, was taken as the sequence between the two PstI sites in Genbank J01783.1. The sequence of the *cat* gene for chloramphenicol resistance was taken from plasmid pCAH56^42^. The sequence of the negative selection cassette *rgnB-*P_*rhaB*_*-relE-tL3* was taken from pSLC-217^24^. The sequence of the R1 *parCMR* partitioning system was taken from Genbank X04268.1. The sequence of the P_*lac*_ promoter was taken from pUC19^43^. The sequence of the P_*araB*_ promoter was taken from plasmid pLA2^42^; a G->A mutation was introduced at position 541 (coordinates according to Genbank AY054373.2) to eliminate a BamHI site. The sequence of the mCherry protein was taken from Genbank AY678264^44^. The sequence of the LacI-93-EGFP protein was deduced from^45^. All proteins were codon optimized for *E. coli* and designed to avoid the following restriction sites: ClaI, XhoI, NotI, PvuI, NciI, NheI, XbaI, HindIII, BamHI, BglII, and SpeI.

### Subcloning of plasmids

Subcloning of plasmids to replace the partitioning system used the unique SpeI and AatII restriction sites in pSLC-295. Briefly, pSLC-295 was cut with SpeI and AatII to remove the R1 *parCMR*, blunted using T4 DNA polymerase, then religated on itself to yield pSLC-298, which carried no partitioning system and served as a negative control for partitioning system function. An R1 *parCMR* fragment with a D170E mutation in *parM* was amplified from pSLC-295 by overlapping PCR using primers 1 to 4, cut with SpeI and AatII, and cloned into the same sites in pSLC-295 to yield pSLC-299. Alp16 *parCMR* (GenBank NZ_ABDW01000017, coordinates 11721–13572) was synthesized (with codon usage for *parM* and *parR* optimized for *E. coli*) with a ParM D160E mutation (Genscript, Hong Kong) and subcloned into the SpeI and AatII sites of pSLC-295 to yield pSLC-300. The ParM D160E mutation was reverted to the wild type sequence by PCR and cloned into the SpeI and PvuI sites of pSLC-300 to yield pSLC-302. Plasmid pSLC-303 (*parC* swap) was created by amplifying the R1 *parC* fragment from pSLC-295 with primers 1 and 11, cutting this PCR product with SpeI and AvrII, and cloning this fragment into pSLC-302 using the same sites to replace the wild type Alp16 *parC* locus. To create pSLC-304, primers 11 to 14 were used to amplify the Alp16 *parMR* locus, introducing a single XbaI site at positions 59 to 64 of *parR* (the introduction of this XbaI site does not result in any amino acid change in the encoded ParR protein; thus this strain is indicated in Fig. 3e as “*parR* sm” (silent mutation)). This PCR fragment was cloned into pSLC-302 using AvrII and AatII, resulting in pSLC-304. To generate pSLC-305 (carrying a ParR truncation mutation), pSLC-304 was cut with XbaI, blunted using T4 DNA polymerase, and religated on itself, introducing a 4 bp frameshift insertion between nucleotide positions 60–61 of the *parR* gene. To generate pSLC-329, a gentamicin resistant plasmid carrying the λ-Red recombinase, pKM208^46^, was digested with BsaI and XmnI to remove the ampicillin gene and blunted with T4 DNA polymerase. A PCR fragment containing the *gen* antibiotic cassette flanked by SphI and NotI was amplified from plasmid pAH152^42^ using primers 50 and 51, digested with these enzymes, blunted with T4 DNA polymerase, and then ligated to the above blunted pKM208 backbone. The ligation product was then transformed into EC958 and screened by colony PCR using primers 52 and 53.

For all subclonings, colony PCR reactions were performed to screen the clones. PCR products with correct product size were sequenced for verification.

### Recombination

The Lambda red recombinase^46,47^ was used to insert the negative selection cassette into plasmid pEC958 directly in strain EC958. Briefly, PCR products of the negative selection cassette *rgnB-*P_*rhaB*_*-relE-tL3* flanked with 50 bp homology arms derived from the desired insertion locus were amplified from pSLC-295 using primers indicated in Table S4. The PCR products were electroporated into EC958 containing the gentamicin resistant plasmid pSLC-329 (which carries the Lambda Red recombinase genes), recovered at 37 °C with shaking at 200 rpm for 2 hours, incubated at room temperature without shaking for 2 hours, then plated onto LB/chloramphenicol plates. Clones resistant to chloramphenicol were restreaked to verify resistance and colony purity, then confirmed by both colony PCR and sequencing of the expected recombination breakpoints.

### Plasmid loss measurement

All strains were streaked out to single colonies on LB/chloramphenicol plates and grown at 37 °C overnight. Single colonies from these plates were then subjected to the following assays for plasmid loss.

1. **Naïve test**. A single colony was inoculated into 3 mL of LB/chloramphenicol and grown at 37 °C with shaking at 200 rpm overnight. The next day, the culture was diluted 1000× into LB broth and grown at 37 °C for 4–6 hours to mid-log phase (OD_600_ = 0.5). Bacteria from 1 mL of this culture were collected by centrifugation at maximum speed and washed once with 100 µL 1 × M9 salts, then resuspended in 100 µL of 1 × PBS. The PBS suspension was titered on M9 glucose and M9 rhamnose plates. The plasmid loss frequency was calculated as the bacterial titer on M9 rhamnose divided by the titer on M9 glucose.
2. **Day zero test**. A single colony was inoculated into 3 mL of LB/chloramphenicol and grown at 37 °C with shaking at 200 rpm overnight. The next day, the culture was adjusted to OD_600_ = 0.5 by dilution with LB broth. Immediately afterward, bacteria from 1 mL of this OD-standardized culture were collected by centrifugation, washed once with 100 μL of 1 × M9 salts, then resuspended in 100 μL of 1 × PBS and titered on M9 glucose and M9 rhamnose plates as above. The plasmid loss frequency was calculated as the bacterial titer on M9 rhamnose divided by the titer on M9 glucose.
3. **Single colony test**. A single colony was re-streaked again to single colonies on LB plates without antibiotic and grown at 37 °C overnight. The next day, these colonies were screened for mCherry expression under a fluorescence dissecting microscope (Olympus MVX10 (which includes GFP and RFP filters) with an Olympus U-LH100HG mercury lamp); a 1–2 mm fluorescent colony (~10^7^ cells) was then scraped from the plate, resuspended in 100 µL of 1 × M9 salts (as a wash), then pelleted by centrifugation and resuspended in 100 μL 1 × PBS and titered on LB/chloramphenicol, M9 glucose, M9 rhamnose plates. Any growth on LB/chloramphenicol was taken as verification that the founding bacterium for the colony did indeed carry the plasmid. The plasmid loss frequency was calculated as the bacterial titer on M9 rhamnose divided by the titer on M9 glucose.
4. **Luria-Delbrück test**. A 1–2 mm colony (~10^7^ cells) was scraped from an M9 glucose/chloramphenicol plate and washed in 100 μL of 1 × M9 salt, followed by resuspension in 100 μL of 1 × PBS. This was titered using 10-fold serial dilution to determine the total number of bacteria and the plasmid-free cell population. A large (2–3 mL) volume of a 10^−7^ dilution into M9 glucose with 0.2% casamino acids was then made; 200 μL of this was aliquoted into each of the 60 wells in the center of a 96-well V-bottom plate (Corning, New York, USA), excluding those near the edge (due to increased evaporation during subsequent culture). Two 96-well plates were used for each strain. Each plate was covered with a loose-fitting lid and incubated at 37 °C with shaking at 200 rpm for about 7 hours. Cultures from 10 of the 60 wells from each of the two plates (20 wells total) were titered to estimate the total number of bacteria after growth. Bacteria from the other 50 wells from each plate (100 wells total) were collected by centrifugation (Eppendorf 5810R, rotor A-4-44, 4000 rpm, 20 min), washed with 200 μL of M9 rhamnose, pelleted by another centrifugation (4000 rpm, 20 min), resuspended in 200 μL of M9 rhamnose, and incubated at 37 °C with shaking at 200 rpm overnight. The bacteria from these 100 wells (2 plates) were again collected by centrifugation, resuspended in 10 μL of M9 rhamnose, and spotted onto M9 rhamnose plates for growth at 37 °C for 2–3 days. The number of wells (out of 100) that grew any colonies was used in the Luria-Delbrück calculation of plasmid loss rate.
5. **Colony patching**. A single colony was inoculated into 3 mL of LB broth without antibiotics and grown at 37 °C with shaking at 200 rpm. This culture was passaged every 24 h by diluting a 3 μl aliquot into 3 ml of fresh LB broth without antibiotics. After one or five passages, an aliquot of 100 μl was serially diluted into PBS and 100 μl of the 1,000,000× dilution was plated on an LB plate without antibiotics and incubated at 37 °C overnight. The next day, 100 to 500 random colonies from this plate were patched onto both LB and LB/chloramphenicol plates to check for colonies which lost chloramphenicol resistance (indicating plasmid loss).

### Verification of plasmid pEC958 loss by PCR and plasmid encoded antibiotic resistance

Single colonies grown on M9 rhamnose plates from the plasmid loss measurement assays were purified by streaking to single colonies on M9 rhamnose plates and then checked by PCR using primers 32 and 54, targeting the plasmid origin *oriV-1*. The absence of a PCR product for *oriV-1* indicated plasmid loss; this was further verified by streaking the same colonies onto LB, LB/kanamycin, and LB/tetracycline plates to ensure sensitivity to both kanamycin and tetracycline.

### Luria-Delbrück calculations

The analysis of the modified Luria-Delbrück data was performed as described for the original fluctuation test^22^, but here, instead of phage resistance leading to bacterial survival, plasmid loss is the “mutation” event that leads to bacterial survival that was measured. Therefore, the plasmid loss rate (P_LR_) is calculated by the following equation:

$${{\rm{P}}}_{{\rm{LR}}}=-\mathrm{ln}\,\frac{({\rm{Y}}-{\rm{Xt}})}{{\rm{Y}}\cdot {\rm{Nt}}}$$

where X_t_ is the number of samples that have plasmid-free cells after growth for time t (7 hours for our assays); Y is the total number of replicate samples tested; and N_t_ is the total number of bacterial cells per sample after growth for time t.

The plasmid loss rate for each strain was plotted as the mean of the loss rates measured in at least 2 independent experiments.

### Transcription start site determination

Transcription start sites for the *parCMR* systems were performed in *E*. *coli* MDS42 cells using the 5′ RACE system for Rapid Amplification of cDNA Ends version 2.0 kit (Thermo Fisher Scientific, Singapore) according to the manufacturer’s instructions, with slight modifications. Briefly, a single colony was inoculated in LB broth with 0.2% of glucose or arabinose and grown at 37 °C at 200 rpm until mid-log phase (OD_600_ ~0.5). RNA was extracted from 10 mL of this culture using the Qiagen RNeasy Mini Kit (Qiagen). 5 μg of total RNA was used as the template for first strand cDNA synthesis using primers 19 and 22 and purified using a S.N.A.P column (Thermo Fisher Scientific, Singapore). 10 µL of the purified cDNA was then dC-tailed by TdT and PCR amplified using the specific nested reverse primer 20 and the abridged anchor primer 21. 10 μL of the PCR product was analyzed on a 1% agarose gel for a single product with the expected band size. 4 μl of the PCR product was cloned into the pCR4 vector (Thermo Fisher Scientific, Singapore) using the TOPO TA Cloning Kit (Thermo Fisher Scientific, Singapore). Clones from the TA cloning were screened by colony PCR using the M13 reverse and M13 forward (−20) primers 24 and 25; for each sample, 8 clones with positive PCR products were sequenced to identify the transcription start site.

### Determination of plasmid copy number

Plasmid copy number (average number of plasmids per bacterial cell) was estimated using quantitative PCR on crude whole DNA prepared from a mid-log phase culture (OD_600_ ~0.6) for strains SLC-202, SLC-726, SLC-729, and SLC-733. 100 μL aliquots from 10-fold serial dilutions of culture were heated at 95 °C for 20 min to lyse the bacteria and release the DNA, then centrifuged at maximum speed in a microcentrifuge for 5 min to pellet the cell debris. The supernatant was used as the DNA template in each qPCR reaction. Each qPCR reaction had a total volume of 20 µL, containing: 1 × KAPA SYBR Fast qPCR master mix (Kapa Biosystems, Wilmington, Mass., USA); 200 nm of each primer; 1 × ROX low reference dye; and 2 µL of the DNA template. qPCR reactions were run using an Applied Biosystems 7500 fast real-time PCR machine with the following protocol: 95 °C for 3 min then 40 cycles of 95 °C for 10 s and 60 °C for 30 s. The experiment was performed with two biological replicates, each biological replicate consisting of three technical replicates. For each strain, two sets of primers were used, one targeting the 16S rRNA housekeeping gene (primers 46 and 47; assumed copy number of 7) on the chromosome of *E*. *coli* and the other targeting the plasmid (primers 48 and 49).

## Electronic supplementary material

Supplemental Information
