# Author Correction: Direct and convenient measurement of plasmid stability in lab and clinical isolates of *E. coli*

**DOI:** 10.1038/s41598-018-23595-w

**Published:** 2018-04-11

**Authors:** Siyi Chen, Mårten Larsson, Robert C. Robinson, Swaine L. Chen

**Affiliations:** 1 Department of Medicine, Yong Loo Lin School of Medicine, National University of Singapore, 1E Kent Ridge Road, NUHS Tower Block, Level 10, Singapore, 119228 Singapore; 2 Institute of Molecular and Cell Biology, A*STAR (Agency for Science, Technology and Research), 61 Biopolis Drive, Singapore, 138673 Singapore; 3 GERMS and Infectious Diseases Group, Genome Institute of Singapore, 60 Biopolis Street, Genome, #02-01, Singapore, 138672 Singapore; 4 Department of Biochemistry, National University of Singapore, Singapore, 117597 Singapore; 5 NTU Institute of Structural Biology, Nanyang Technological University, 59 Nanyang Drive, Singapore, 636921 Singapore; 6 Research Institute for Interdisciplinary Science, Okayama University, Okayama, 700-8530 Japan; 7 Present Address: Department of Medical Biochemistry and Microbiology, Uppsala University, SE-751 23 Uppsala, Sweden

Correction to: *Scientific Reports* 10.1038/s41598-017-05219-x, published online 06 July 2017

The original version of this Article omitted an affiliation for Robert C. Robinson. The correct affiliations are listed below:

Institute of Molecular and Cell Biology, A*STAR (Agency for Science, Technology and Research), 61 Biopolis Drive, Singapore, 138673, Singapore.

Department of Biochemistry, National University of Singapore, Singapore, 117597, Singapore.

NTU Institute of Structural Biology, Nanyang Technological University, 59 Nanyang Drive, Singapore, 636921, Singapore.

Research Institute for Interdisciplinary Science, Okayama University, Okayama 700-8530, Japan.

This has now been corrected in the HTML and PDF versions of this Article.

