# Supplementary material for: Direct and convenient measurement of plasmid stability in lab and clinical isolates of E. coli
Source: Sci Rep. 2017 Jul 6;7:4788. doi: 10.1038/s41598-017-05219-x (PMC5500522; doi:10.1038/s41598-017-05219-x)
Supplement: Supplementary file 1 — Supplemental Information [file 41598_2017_5219_MOESM1_ESM.pdf]

Direct and convenient measurement of plasmid stability in lab and clinical isolates of *E. coli*

Supplementary Information

Siyi Chen, Mårten Larsson, Robert C. Robinson, and Swaine L. Chen

Supplementary Text

Genetic verification of the function of components of the Alp16 *parCMR* system

To further demonstrate the power of this system for understanding novel plasmid loci contributing to stability, we replaced the R1 *parCMR* system with a predicted plasmid segregation system (Alp16 *parCMR*) from a very distantly related bacterium, *Clostridium perfringens* (Figure 1c in the main text). While the *parM* and *parR* protein coding genes could be re-encoded to use codons preferred by *E. coli*, by analogy to the R1 system, *parC* is predicted both to mediate transcription of *parM* and *parR* and to contain the specific DNA operator sequences required for ParR binding [1]. We thus first verified that the unmodified *C. perfringens parC* indeed mediated transcription in *E. coli* by 5'-RACE in a position similar to that reported for the R1 *parC* (Figure S3 and Table S2). By the Luria-Delbrück test, we found that the *C. perfringens* Alp16 *parCMR* mediated nearly 1-log better plasmid maintenance than pSLC-298 (Δ*parCMR*) (Figure 3e in the main text). As expected, introduction of an ATP binding site mutation in ParM (homologous to that used for the R1 ParM) resulted in stability very close to pSLC-298. Furthermore, measurement of stability in a matched pair of plasmids, in which an XbaI site was introduced to facilitate creation of a frameshift mutation in *parR*, showed that disruption of *parR* also gave a ~0.5 log reduction in plasmid stability (Figure 3e in the main text). We again tested the more convenient single colony assay; the results were qualitatively similar, though we now observed some quantitative variation in stability values. Interestingly, the difference in stability for plasmids carrying functional and nonfunctional systems was in all cases measured to be greater with the single colony assay. We suspected that the exaggerated effect may be due to higher growth rates of plasmid-free cells, which would affect the single colony but not the Luria-Delbrück assay. Indeed, we found that strains carrying pSLC-300 (with a D160E ParM mutation) grew slowly relative to plasmid-free cells (Figure S1b) and also had the greatest discrepancy between the single colony and Luria-Delbrück tests (Figure 3e in the main text). While the quantitative correspondence between the convenient single colony and Luria-Delbrück tests breaks down in the context of different growth rates, it would still be a simple and useful screening test for detecting impaired plasmid stability. Overall, this data argues strongly that the Alp16 *parCMR* is functional and operates via a similar mechanism to that of the R1 *parCMR* system. Thus, this synthetic plasmid system provides a promising platform for further studies of arbitrary plasmid maintenance systems.

Discussion of issues affecting expression control for heterologous *parCMR* systems

First, transcriptional signals are thought to be incorporated into the *parC* locus, and might be tightly intertwined with the ParR binding sites, which cannot be changed. Second, codon bias can influence final protein concentrations. Third, translational coupling between ParM and ParR may vary in efficiency and mechanism, especially when codons are optimized to the *E. coli* preference. Fourth, feedback regulation of transcription may be somewhat host-dependent; the model of ParR repressing the *parC* promoter in a negative feedback loop might also be variably effective in a different host. Finally, protein stability is also likely to be different, which may affect the optimal levels of transcription and translation. The latter is particularly difficult to predict, since mechanisms and critical concentrations for assembling different ParM filaments [2, 3] are different and may depend on plasmid size and host cell characteristics (such as size, ATP levels, or molecular crowding, among other parameters) [4-6]. Among these, we attempted only to control the second (we optimized the codon bias of *parM* and *parR* for *E. coli*) and third (we maintained the 1 bp overlap of the stop codon of *parM* with the start codon of *parR*). Interestingly, we found that Alp16 *parC*, with no modifications, functions to drive transcription of *parM*

and *parR* in *E. coli*. By replacing the Alp16 *parC* with the R1 *parC*, not only was plasmid maintenance less faithful than a plasmid with no segregation system (Figure 3e in the main text), but growth rate was severely impacted as well. Loss of segregation function (to the level of a knockout such as pSLC-298) is expected due to the mismatch between R1 *parC* and Alp16 ParR. The more severe segregation phenotype, combined with the reduced growth rate, leads us to suspect that the R1 *parC* is driving expression levels of ParM and ParR beyond those effective for segregation and also beyond those easily tolerated by the cell. This implies that, when Alp16 *parM* and *parR* are being driven by Alp16 *parC*, appropriate negative feedback regulation may be occurring. An alternative to this conclusion is that Alp16 *parC* acts as a promoter in *E. coli* with no regulation by ParR binding, and the expression level of the unregulated Alp16 *parC* happens to be in the range that enables segregation function; but we feel this possibility is less likely. Finally, while we did not measure protein stability, clearly the increased plasmid stability conferred by Alp16 *parCMR*, coupled with our targeted mutations of *parM* and *parR*, argues that ParM and ParR protein stabilities are sufficient for function. Indeed, the fact that Alp16 *parCMR* only provides a 10× increase in stability, compared with the 100× provided by R1 *parCMR*, may be due to suboptimal expression arising from any of these issues.

Supplementary Figures and Tables

a

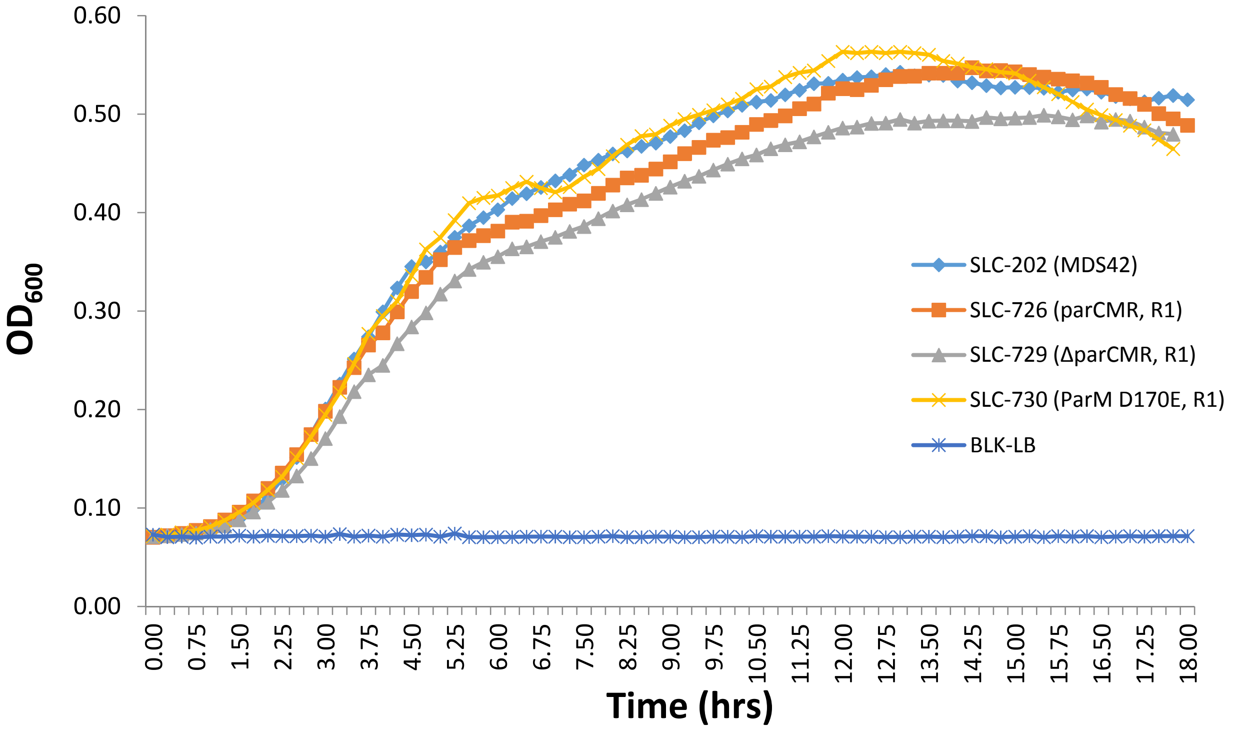

b

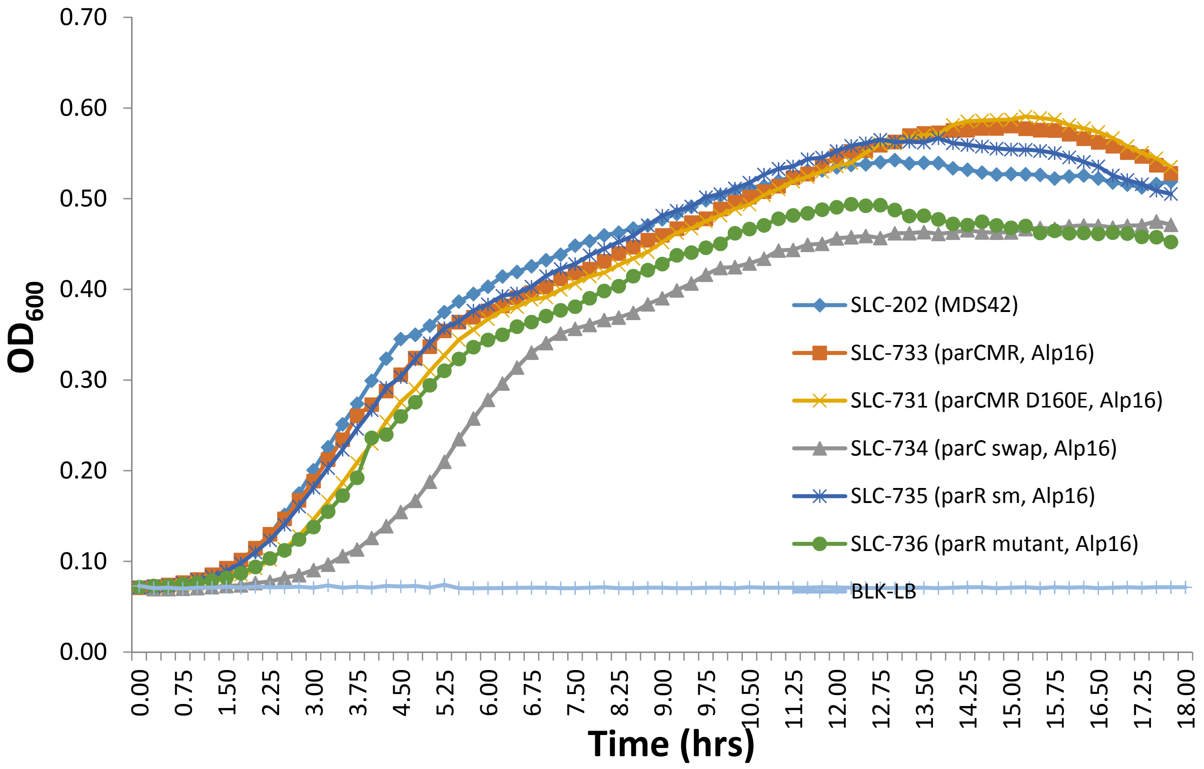

c

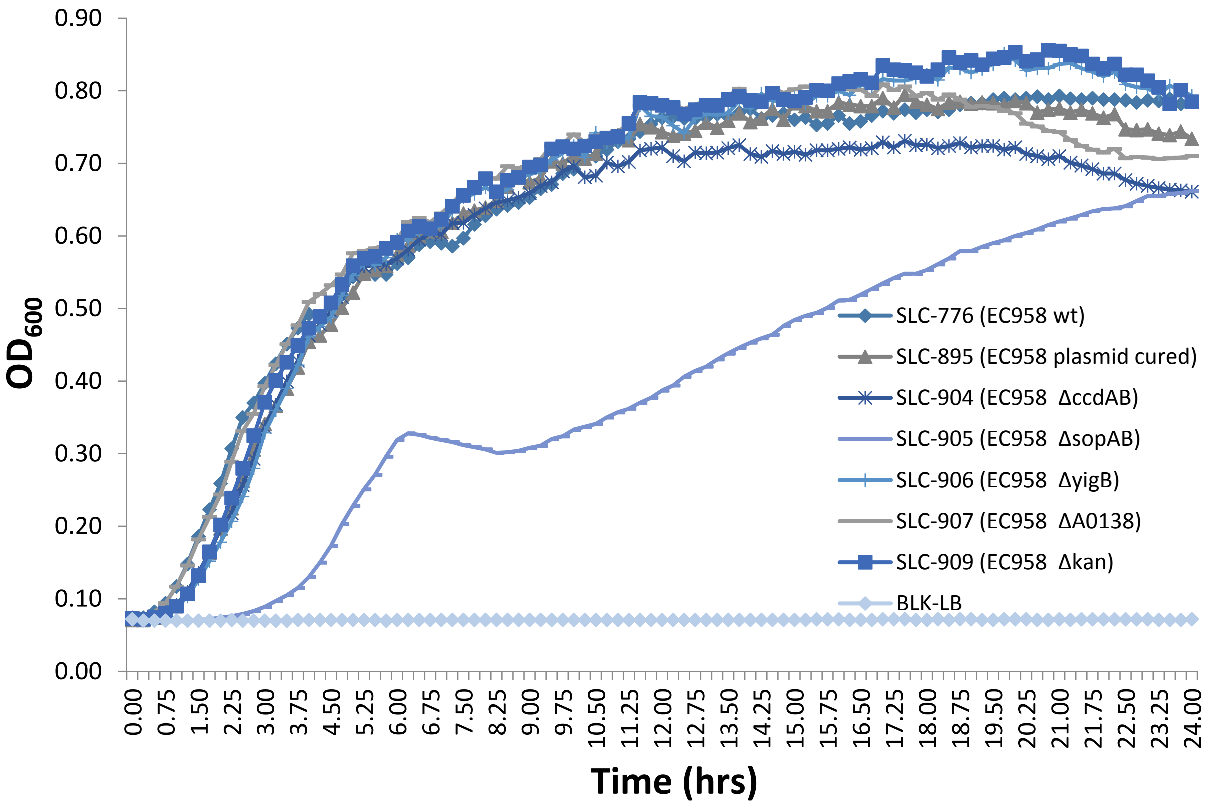

Figure S1. Growth curves of various mutant strains as compared to the wild type strain in LB medium. (a) MDS42 strains carrying plasmids bearing the R1 *parCMR* system or its mutants. (b) MDS42 strains carrying plasmids bearing the Alp16 *parCMR* system or its mutants. (c) EC958 strains carrying the wt or mutant pEC958 plasmids.

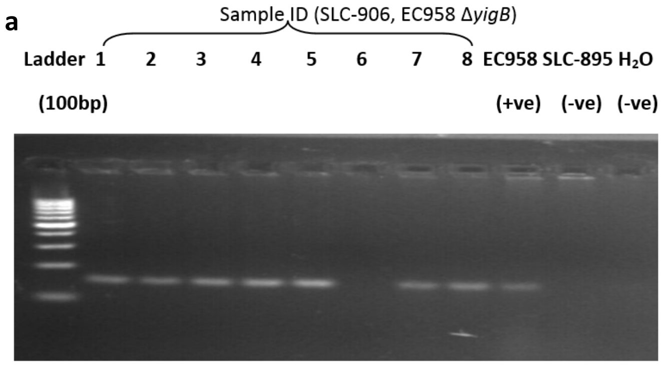

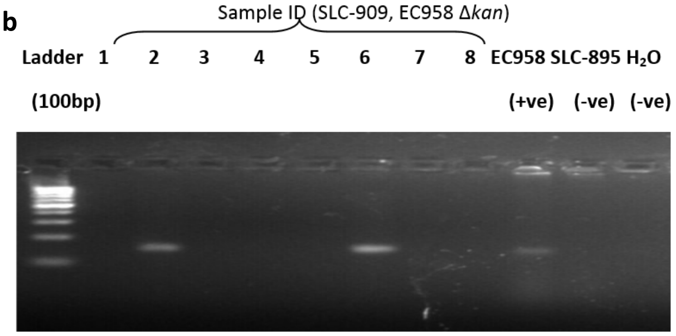

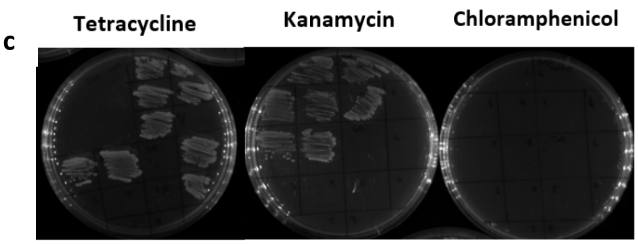

d

Phenotype/Genotype	SLC-906(EC958 Δ*yigB*)							
	1	2	3	4	5	6	7	8
Tetracycline	+	+	+	+	+	-	+	+
Kanamycin	+	+	+	+	+	-	+	+
Chloramphenicol	-	-	-	-	-	-	-	-
PCR (*oriV-1*)	+	+	+	+	+	-	+	+
pEC958 plasmid	+	+	+	+	+	-	+	+

Phenotype/Genotype	SLC-909(EC958 Δ*kan*)							
	1	2	3	4	5	6	7	8
Tetracycline	-	+	-	-	-	+	-	-
Kanamycin	-	-	-	-	-	-	-	-
Chloramphenicol	-	-	-	-	-	-	-	-
PCR (*oriV-1*)	-	+	-	-	-	+	-	-
pEC958 plasmid	-	+	-	-	-	+	-	-

Figure S2. Verification of true plasmid loss by PCR and plasmid encoded antibiotic resistances. Strains that grew on rhamnose could have either lost the entire plasmid or inactivated the negative selection cassette in some other fashion while retaining all or part of the plasmid. We used a PCR to the functional origin (*oriV-1*) of pEC958 [7] (expected size 150bp) and sensitivity to antibiotics with plasmid-encoded resistance genes (kanamycin and tetracycline; the chloramphenicol resistance gene is not natively present on pEC958 but is introduced with and tightly linked to the negative selection cassette) to distinguish between these two possibilities. A negative *oriV-1* PCR and loss of resistance to these antibiotics was interpreted as loss of the entire plasmid. (a) PCR results for the *oriV-1* locus for colonies derived from SLC-906 (EC958, Δ*yigB*) strains that grew on rhamnose and controls. (b) PCR results for the *oriV*-1 locus for colonies derived from SLC-909 (EC958, Δ*kan*) strains that grew on rhamnose and controls. (c) Patch tests for antibiotic resistance on colonies derived from SLC-906 (EC958, Δ*yigB*) and SLC-909 (EC958, Δ*kan*) that grew on rhamnose. The antibiotic tested is indicated above each plate. (d) Summary of the results from these assays, demonstrating perfect concordance between the *oriV-1* PCR and the antibiotic resistance patch test among these 16 colonies derived from SLC-906 (top) and SLC-909 (bottom).

```
                    +1 parC
4900   AAGCGGCCGCACTAGTAAAAAATAACTCCTTTCATCTAGGAAAGTACACTAAATCCTAAATAGAAAGAAGT   4969

4970   TAAAATTTGCTAATATTACATTTCTGTTAAAAAGTCAATATATTTAATTGACTTTTTTCAATAGCTTTGA   5039

5040   TATCATAAAGCTATGAAAAGTTAATATTTCAAATAGAACTTTGTTCTATTTCGGATTTTAGTTCATCCAT   5109

5110   TAAGTTTTCAAAGACGCCAATCGAAGTAAACTTAATGGATTTTTCCTTTTCCGTACTATTTAATTTAATT   5179

5180   GAGTTAATAATACCATGTTAAAAATAATTTTTCAAGAGAGTAAAATTATAGAAAATTTTACTCTCTTTTT   5249

5250   TTATTAATTTCAATATATGTATATACTGAATATATACATATATTGAAATTAATAAAATATAATTATATAT   5319

5320   ACTTAGTATATACAAATTATATTTAGTATATATATAGTATATACTAAAGTAAGAAAATTAAAGAGAATAA   5389

5390   TAGTATTTCGAAGAAAGAATAGTATAAACTCAGTATATACATAATTAGGGGAGTTGTTTTAGTATATAT   5459
                        parC start site                  +1 parM
5460   CTAGTATATACTAAATTTATAAAAGATAATTAATTTTGAAAGGAGCATTAAAATGCTGAAACTGGGTATC   5529

5530   GACCTGGGTAATGGCTACACCAAATTCAAAGGCTCCAAATTCGCGTCAAAAACCAAAGTGGGCCGTCTGG   5599
```

Figure S3. Transcription start site of Alp16 *parCMR* in pSLC-302 in MDS42 host cells. Alp16 *parC* sequence and partial sequence of Alp16 *parM* is shown in the graph. Transcription of Alp16 *parCMR* in MDS42|pSLC-302 cells is found to start from position +563 of *parC*, 34bp upstream of *parM*. Nucleotide coordinates are based on the sequence of pSLC-302.

Table S1. Plasmid copy number by qPCR

Strain & Plasmid ID	Plasmid Copy Number
MDS42	ND*
MDS42|pSLC-295 (R1 *parCMR*)	7
MDS42|pSLC-298 (Δ*parCMR*)	7
MDS42|pSLC-302 (Alp16 *parCMR*)	8-10

*ND, not detected.

Table S2. Transcription start site of *parCMR* system in different plasmids in *E. coli*. The length of the R1 *parC* is 182 bp. The length of the Alp16 *parC* is 596 bp.

Strain ID	Bacterial Strain and Plasmid	Partitioning System	Transcription Start Site	
			5'-RACE Assay	From literature
SLC-726	MDS42|pSLC-295	*parCMR* (R1)	+115 in R1 *parC*	+115 in R1 *parC* [8]
SLC-733	MDS42|pSLC-302	*parCMR* (Alp16)	+563 in Alp16 *parC*	Not available

Table S3. Bacterial strains and plasmids used in this study and their source

Strain ID	Bacterial Strain and Plasmid	Plasmid *par* Locus Relevant Genotype	Plasmid Source and Origin
SLC-202	MDS42	-	
SLC-726	MDS42|pSLC-295	*parCMR* (R1)	This study, by gene synthesis (GenScript, Piscataway, NJ, USA)
SLC-729	MDS42|pSLC-298	Δ*parCMR* (R1)	Subcloning from pSLC-295
SLC-730	MDS42|pSLC-299	ParM D170E (R1)	Subcloning from pSLC-295
SLC-733	MDS42|pSLC-302	*parCMR* (Alp16)	This study, by gene synthesis (GenScript) and subcloning
SLC-731	MDS42|pSLC-300	ParM D160E (Alp16)	Subcloning from pSLC-302
SLC-734	MDS42|pSLC-303	*parC*(R1) –*parMR* (Alp16)	Subcloning from pSLC-302
SLC-735	MDS42|pSLC-304	*parCMR* (Alp16), w/ XbaI on *parR* (silent mutation)	Subcloning from pSLC-302
SLC-736	MDS42|pSLC-305	*parCMR* (Alp16), w disrupted *parR*	Subcloning from pSLC-304
SLC-896	CFT073|pSLC-295	*parCMR* (R1)	This study
SLC-897	CFT073|pSLC-298	Δ*parCMR* (R1)	This study
SLC-898	UTI89|pSLC-295	*parCMR* (R1)	This study
SLC-899	UTI89|pSLC-298	Δ*parCMR* (R1)	This study
SLC-904	EC958|pSLC-341	pEC958A, *ccdA&ccdB::cat-rgnB*-P*rhaB-relE-tL3*	This study
SLC-905	EC958|pSLC-342	pEC958A, *sopA&sopB::cat-rgnB*-P*rhaB-relE-tL3*	This study
SLC-906	EC958|pSLC-343	pEC958A, *yigB::cat-rgnB*-P*rhaB-relE-tL3*	This study
SLC-907	EC958|pSLC-344	pEC958A, *A0138::cat-rgnB*-P*rhaB-relE-tL3*	This study
SLC-908	EC958|pSLC-329	pKM208 *amp*::*gent*	This study
SLC-909	EC958|pSLC-345	pEC958A, *aac(6')-Ib-cr::cat-rgnB*-P*rhaB-relE-tL3*	This study
SLC-895	EC958, plasmid pEC958A cured		This study

Table S4. Primers used in this study

Primer ID	Sequence (5' – 3')	Position	Reference	Note
1	CGGCCTTTCTGTTATCCGAA	pSLC-295/pSLC-302 +4719	This study	
2	TAAAGCCTGGGGTGCCTAAT	pSLC-295-7077/pSLC-302 -6869	This study	
3	GTGCCCCCGAGTTCTATAA	-	This study	
4	TTATAGAACTCGGGGGCAC	-	This study	
5	GCGCAATCACTTTCGTCTCT	pSLC-295/pSLC-302 +4668	This study	
6	GTGACCTTCAGTTTTGCCGT	pSLC-295 -7308/pSLC-302 -7100	This study	
7	ACCGAAGCAATGAATGAAGCA	pSLC-295 +5842	This study	
8	GTTCTGCGCTTGTCCATCAT	pSLC-295 -6079	This study	
9	GTATCGACCTAGGTAATGGC	pSLC-302 +5525	This study	
10	GCAGGCAGATTTCGTAGTCC	pSLC-302-5737	This study	
11	TGGTGTAGCCATTACCTAGGTCGATACCCAGTTTCAG CATTTATAAAACTCCTTATGGTG	-	This study	
12	CGCCCAGTTCGTCAATGTAGTCTAGAATTTCTTTATCA CTTTTGGTGT		This study	pSLC-297 ParR XbaI Rev
13	ACACCAAAAGTGATAAAGAAATTCTAGACTACATTGA CGAACTGGGCG		This study	pSLC-297 ParR XbaI Fwd
14	ATGCTGAAACTGGGTATCGA	pSLC-302 +5512	This study	
15	GCTGCCTCCTGTGTGAAATT	pSLC-295 -7146/pSLC-302 -6938	This study	
16	GTTTGACACAATCGCGGAGA	pSLC-302 -6246	This study	
17	CTTTCAGCTGTTCCGCGATT	pSLC-302-6618	This study	
18	AACATTACGCAATGGGATAATCT	pSLC-302 +6010	This study	
19	GAAGCTGTTCGGGCTAATGT	pSLC-295 -5193	This study	5'-RACE assay, cDNA synthesis primer for pSLC-295
20	TAATTGTTCCGTCGCTTTCC	pSLC-295 -5167	This study	5'-RACE assay, nested reverse primer for PCR amplification of cDNA sample from pSLC-295
21	GGCCACGCGTCGACTAGTACGGGIIGGGIIGGGIIG	-	5'-RACE kit	5'-RACE assay, abridge anchor (forward) primer for PCR amplification of all cDNA samples
22	GGAGCCTTTGAATTTGGTGT	pSLC-302 -5565	This study	5'-RACE assay, cDNA synthesis primer for pSLC-302
23	TCACCGACAATGTACGTGGT	pSLC-302 -5678	This study	5'-RACE assay, nested reverse primer 2 for pSLC-302
24	CAGGAAACAGCTATGAC	M13 reverse	TOPO TA Cloning Kit	Forward primer for colony PCR and sequencing

Primer ID	Sequence (5' – 3')	Position	Reference	Note
25	GACCGGCAGCAAAATG	M13 forward (-20)	TOPO TA Cloning Kit	Reverse primer for colony PCR and sequencing
26	GAGGGGCGGCCAAGGGCGCCAGCCCTTGGACGTCCC CCTCGATGGAAGGGGCTGATAGAAACAGAAGCCACT	pEC958+104760-pSLC-295-chlor	This study	
27	TGCCTTTAAGCGTGCATAATAAGCCCTACACAAATTG GGAGTTAGACATCTTCTTCGTCTGTTTCTACTGGTATT	pEC958-105459-pSLC-295-relE	This study	
28	ATTAATGAGGCTCCGGGTTC	pEC958+ 104724	This study	Primer used for colony PCR and also sequencing of SLC-909
29	GCCCTACACAAATTGGGAGT	pEC958-105437	This study	Primer used for colony PCR and also sequencing of SLC-909
30	ATGAAGCAGCGTATTACAGTGACAGTTGACAGCGAC AGCTATCAGTTGCTGCTGATAGAAACAGAAGCCACT	pEC958+408-pSLC-295-chlor	This study	
31	TTATATTCCCCAGAACATCAGGTTAATGGCGTTTTTGA TGTCATTTTCGCTTCTTCGTCTGTTTCTACTGGTATT	pEC958-933-pSLC-295-relE	This study	
32	AGTCGGATCCATACGAAACG	pEC958+200	This study	Primer used for colony PCR, sequencing of SLC-904, checking true plasmid loss
33	ACACTGCCGGTACCATGACT	pEC958-985	This study	Primer used for colony PCR and also sequencing of SLC-904
34	ATGAAACTCATGGAAACACTTAACCAGTGCATAAACG CTGGTCATGAAATGCTGATAGAAACAGAAGCCACT	pEC958+3587-pSLC-295-Chlor(FWD):	This study	
35	TCAGGGTGCTGGCTTTTCAAGTTCCTTAAGAATGGCCT CAATTTTCTCTATTCTTCGTCTGTTTCTACTGGTATT	pEC958-5994-pSLC-295-relE(REV)	This study	
36	CAGTTCGCTCGCTATGCTC	pEC958+3644	This study	Primer used for colony PCR and also sequencing of SLC-905
37	CAGTGGGCCCAGAGAGAATA	pEC958-6094	This study	Primer used for colony PCR and also sequencing of SLC-905
38	ATGAGGAAATATATTCCACTGGTATTATTTATCTTTTC ATGGCCGGTATTGCTGATAGAAACAGAAGCCACT	pEC+67699-pSLC-295-Chlor(FWD)	This study	
39	TTATTTACGGTGTCTCCATATCCAGGGGGGAACCGGA TCAGCATCTGACCTTCTTCGTCTGTTTCTACTGGTATT	pEC-68160-pSLC-295-relE(REV)	This study	
40	TGGAGCTGCATGACAAAGTC	pEC958+67599	This study	Primer used for colony PCR and also sequencing of SLC-906

Primer ID	Sequence (5' – 3')	Position	Reference	Note
41	CCTGTTTTGTTTTCGCCATT	pEC958-68224	This study	Primer used for colony PCR and also sequencing of SLC-906
42	ATGGAACTGAAGTGGACCAGTAAGGCGCTTTCTGATT TGTCGCGGTTATTGCTGATAGAAACAGAAGCCACT	pEC+117295-pSLC-295-chlor(FWD)	This study	
43	CTACCTGTTTTCTCGTGTGTGCCACAGACGCAATACAT AAATAGTCTGGCTTCTTCGTCTGTTTCTACTGGTATT	pEC-117570-pSLC-295-relE(REV)	This study	
44	TGTGACATCCGGACAGGTTA	pEC958+117205	This study	Primer used for colony PCR and also sequencing of SLC-907
45	CGTTCAGCGGTAATTTTCGT	pEC958-117713	This study	Primer used for colony PCR and also sequencing of SLC-907
46	AACGCGAAGAACCTTAC	V6 (16s rRNA, *E. coli*)	This study	*E. coli* 16s rRNA fwd primer used in qPCR for plasmid copy number
47	CCTTTGAGTTCCCGGCC	R2 (16s rRNA, *E. coli*)	This study	*E. coli* 16s rRNA rev primer used in qPCR for plasmid copy number
48	GGGGATGGGTAAGGTGAAAT	pSLC-295+2457 fwd	This study	Plasmid fwd primer used in qPCR for plasmid copy number
49	CGAAGTGATCTTCCGTCACA	pSLC-295-2821 rev	This study	Plasmid rev primer used in qPCR for plasmid copy number
50	AATGATACGGCGAGACCTGGCCGGATCTGCCGGCGG CCGC	pAH152-BsaI-pKM208-NotI	This study	Forward primer for amplifying *gen* from pAH152 [9]
51	CTGCAGGCATGCAAGCTT	pAH152-sphI+5bp	This study	Reverse primer for amplifying *gen* from pAH152 [9]
52	TCACCGTAATCTGCTTGCAC	pAH152+2698	This study	Primer used for colony PCR of pSLC-329
53	CATCATTCGCACATGTAGGC	pAH152-3052	This study	Primer used for colony PCR of pSLC-329
54	AAAAATCAGCGCGCAAATAC	pEC-349	This study	Primer used for checking true plasmid loss

Supplementary References

1. Breuner, A., Jensen, R.B., Dam, M., Pedersen, S. & Gerdes, K. The centromere-like parC locus of plasmid R1. *Mol Microbiol* **20**, 581-592 (1996).
2. Gunning, P.W., Ghoshdastider, U., Whitaker, S., Popp, D. & Robinson, R.C. The evolution of compositionally and functionally distinct actin filaments. *J Cell Sci* (2015).
3. Jiang, S. et al. Novel actin filaments from Bacillus thuringiensis form nanotubules for plasmid DNA segregation. *Proc Natl Acad Sci U S A* **113**, E1200-1205 (2016).
4. Ebersbach, G. & Gerdes, K. Plasmid segregation mechanisms. *Annu Rev Genet* **39**, 453-479 (2005).
5. Polka, J.K., Kollman, J.M., Agard, D.A. & Mullins, R.D. The structure and assembly dynamics of plasmid actin AlfA imply a novel mechanism of DNA segregation. *J Bacteriol* **191**, 6219-6230 (2009).
6. Rivera, C.R., Kollman, J.M., Polka, J.K., Agard, D.A. & Mullins, R.D. Architecture and assembly of a divergent member of the ParM family of bacterial actin-like proteins. *J Biol Chem* **286**, 14282-14290 (2011).
7. Phan, M.D. et al. Molecular characterization of a multidrug resistance IncF plasmid from the globally disseminated Escherichia coli ST131 clone. *PLoS One* **10**, e0122369 (2015).
8. Gerdes, K. & Molin, S. Partitioning of plasmid R1. Structural and functional analysis of the parA locus. *J Mol Biol* **190**, 269-279 (1986).
9. Haldimann, A. & Wanner, B.L. Conditional-replication, integration, excision, and retrieval plasmid-host systems for gene structure-function studies of bacteria. *J Bacteriol* **183**, 6384-6393 (2001).
